# An Acute Subdural Hemorrhage Due to a Left Supraclinoid Internal Carotid Artery Aneurysm Rupture Without a Subarachnoid Hemorrhage

**DOI:** 10.7759/cureus.22462

**Published:** 2022-02-21

**Authors:** Khalid T Alghamdi, Luma Qutub, Wed T Alghamdi, Abdulrahman Alshamy, Hussam Kutub

**Affiliations:** 1 College of Medicine, King Saud bin Abdulaziz University for Health Sciences, King Abdullah International Medical Research Centre, Jeddah, SAU; 2 College of Medicine, Batterjee Medical College, Jeddah, SAU; 3 Faculty of Medicine, King Abdulaziz University, Jeddah, SAU; 4 Neuroradiology, King Abdulaziz Medical City, Ministry of National Guard Health Affairs, Jeddah, SAU; 5 Consultant Neurosurgery, King Abdulaziz Medical City, Ministry of National Guard Health Affairs, Jeddah, SAU

**Keywords:** aneurysm rupture, pure subdural hematoma, subdural hematoma following aneurysm ruptured, brain aneurysm, subdural hematoma

## Abstract

Acute subdural hemorrhage (SDH) is a rare complication that can occur after a spontaneous intracranial aneurysmal rupture. It is commonly associated with a subarachnoid and/or an intracerebral hemorrhage but rarely occurs as an SDH alone. A 52-year-old female presented to our institution with a severe headache and third cranial nerve palsy. A computed tomography (CT) scan revealed acute left SDH, without a subarachnoid hemorrhage (SAH), and a computed tomography angiogram (CTA) and cerebral angiography demonstrated the presence of a left supraclinoid aneurysm pointing towards the cavernous sinus. Endovascular occlusion of the aneurysm was performed using a flow diverter. A follow-up CT scan revealed a resolved SDH. In similar situations, vascular imaging, such as CTA and cerebral angiography, is required to assess the cerebral vasculature. This case report describes a patient presenting with the sudden onset of a severe headache associated with a cranial nerve palsy and a brain CT scan showing an acute SDH in the absence of trauma or an anticoagulation history. The treating physician should be highly vigilant of the possibility of a ruptured intracranial aneurysm as the underlying SDH etiology.

## Introduction

Acute subdural hemorrhage (SDH) is a well-known complication following head trauma and may cause a sudden loss of consciousness and neurological deficits in a previously healthy individual. A ruptured aneurysm of the internal carotid artery (ICA)-posterior communicating artery (Pcom) is the most common site for an associated uncommon spontaneous acute SDH [1]. Intracranial aneurysmal rupture is usually associated with subarachnoid hemorrhage (SAH). Some patients can present with a combination of SAH with intracerebral hemorrhage (ICH) and/or intraventricular hemorrhage (IVH) [1]. Occasionally, patients can present with SDH with SAH [2]. However, SDH alone following an intracranial aneurysmal rupture without SAH rarely occurs [3,4]. We present the case of a patient with an SDH following a supraclinoid ICA aneurysmal rupture without a SAH.

## Case presentation

**Figure 1:**
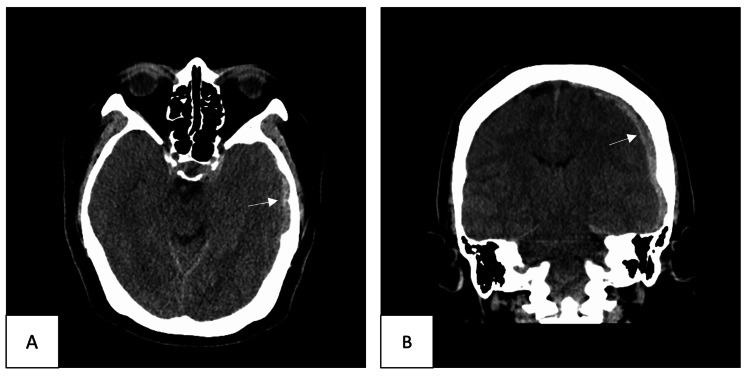
Axial (A) and coronal (B) brain CT images showed a large left acute subdural hematoma with a mild midline shift without any evidence of subarachnoid bleeding

A 52-year-old female on hypertensive medications presented to the Emergency Department (ED) with a headache for three days mainly involving the left side of the head and left eye. The patient was suspected of presenting with migrainous symptoms and was managed with pain relief treatment and metoclopramide. After three days, the patient returned to the ED with a severe headache, double vision and nausea, and vomiting. She had no history of loss of consciousness, seizure, or trauma and had not been treated with an anticoagulant. On neurological examination, she was awake and alert with a Glasgow Coma Scale (GCS) score of 15/15 and was without a motor deficit, except for left third cranial nerve palsy; therefore, we consulted an Ophthalmology team. A brain computed tomography (CT) was performed and showed a large left acute subdural hematoma (Figure 1).

**Figure 2:**
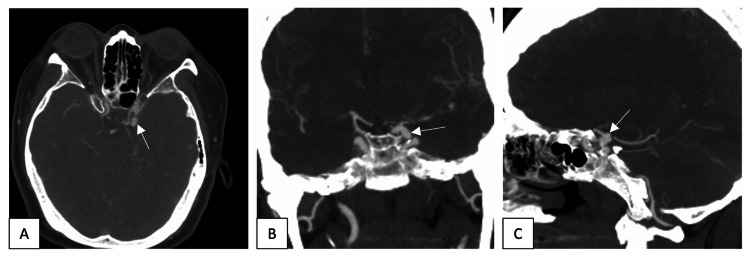
Axial (A), coronal (B), and sagittal (C) CT angiograms showed a left supraclinoid internal carotid artery multi-lobular aneurysm

**Figure 3:**
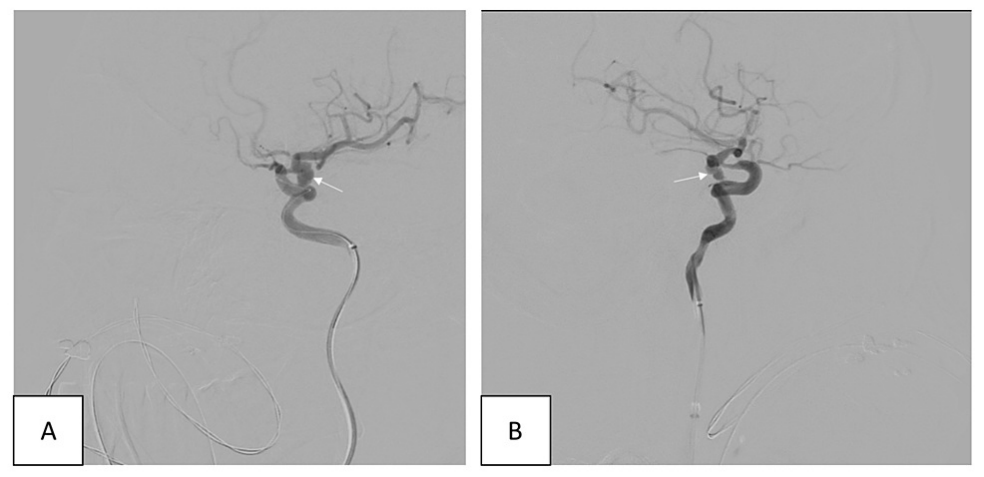
Frontal (A) and Lateral (B) views of a left internal carotid artery (LICA) aneurysm pointing laterally and inferiorly towards the cavernous sinus

Magnetic resonance imaging (MRI) was performed later and confirmed the subdural hematoma and absence of subarachnoid bleeding. A CT angiogram (CTA) showed a left supraclinoid ICA multilobular aneurysm measuring 8 × 6 × 8.5 mm in the anteroposterior, transverse, and craniocaudal dimensions (Figure 2), pointing toward the cavernous sinus and presenting with a wide neck and dysplastic supraclinoid segment (Figure 3).

**Figure 4:**
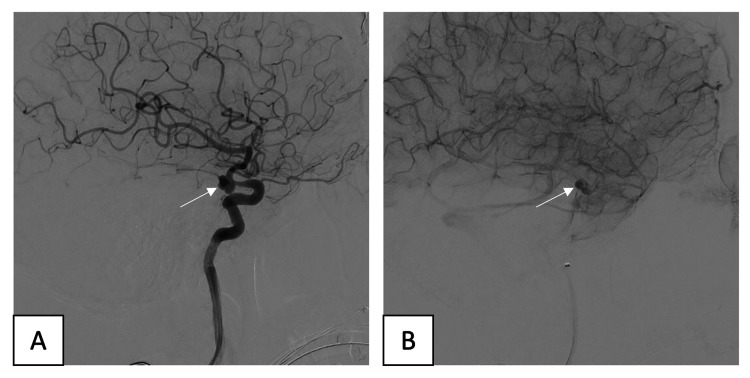
Early (A) and delayed (B) phases after deployment of a flow diverter shows contrast stagnation in the aneurysm

The best therapeutic management was determined to be endovascular management with embolization using a flow diverter (Figure 4). The patient was prepared the day before with loading doses of ticagrelor and aspirin. The diagnostic examination confirmed the wide neck-lobulated aneurysm in the supraclinoid segment of the ICA. The aneurysm pointed laterally, posteriorly, and inferiorly, and extended into the cavernous sinus with the associated underlying dysplastic and narrowed segment in the cavernous portion of the ICA. The patient was treated with 5000 IU of heparin.

**Figure 5:**
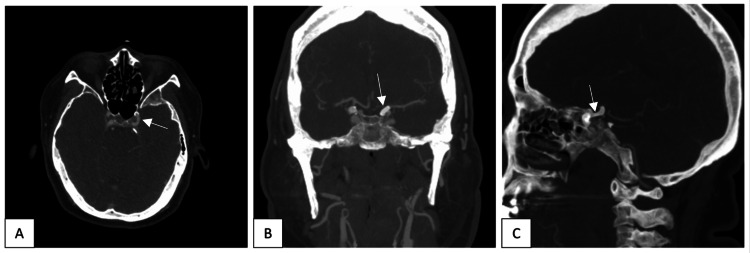
Axial (A), coronal (B), and sagittal (C) CT angiograms post-flow diverters insertion with no evidence of subdural hemorrhage

Slow progressive deployment of a flow diverter (Pipeline™ Flex Embolization Device with Shield Technology™; Medtronic, Minneapolis, MN, USA) started from the distal LICA to the cavernous segment of LICA, which resulted in a significant flow reduction in the aneurysmal sac with contrast stagnation in the delayed phase. No complications occurred during or immediately after the procedure. The patient was discharged after five days of aspirin and ticagrelor. A follow-up brain CTA showed SDH regression and clinical improvement (Figure 5).

## Discussion

The presence of a subdural hematoma without a subarachnoid hemorrhage due to an intracranial aneurysm is an extremely rare occurrence [3,4]. In this case report, we present a case of an SDH alone without any radiological evidence of a SAH. Five mechanisms have been reported in the literature that result in a subdural space hemorrhage: (I) adhesion of the aneurysm to the arachnoid membrane due to minimal successive hemorrhages, (II) extension and then erosion of the aneurysm in the cavernous sinus, (III) laceration of the arachnoid membrane due to high-pressure hemorrhage, (IV) an ICH that tears the brain tissues and arachnoid membrane, and (V) possibility of an aneurysm in the subdural carotid artery directly resulting in a subdural hematoma [1,2]. Clinically, our patient initially presented with a minor headache, which may explain the minimal hemorrhage that led to an aneurysmal adhesion to the arachnoid membrane. The second headache with a third cranial nerve palsy was severe and probably due to a ruptured aneurysm with extension into the subdural space. In addition, the patient denied any history of trauma or anticoagulant intake that could have increased the suspicion of an intracranial aneurysm, especially with the presence of a third nerve palsy. However, the rarity of this condition plays a role in delaying the diagnosis of patients in multiple cases [3-5].

## Conclusions

A high index of suspicion is necessary to diagnose a ruptured ICA aneurysm in a patient with a nontraumatic SDH, especially in the absence of an SAH. Vascular imaging should be performed including a CTA, MRA, or cerebral angiogram to evaluate the entire cerebral vasculature. Furthermore, in the presence of an ICA aneurysm, endovascular management can be performed. Moreover, reporting a similar case or performing a cohort study is important to have a better understanding of the condition and to increase the index of suspicion of healthcare practitioners.
